# Predictive functional profiling of 16S rRNA genes amplicons reveals bioremediation and sulfur metabolism capacity in thermophilic hot spring bacteriomes

**DOI:** 10.1038/s41598-026-50048-6

**Published:** 2026-05-05

**Authors:** Mohamed Ismaeil, Ali M. Saeed, Samah A. Donia, Wael S. El-Sayed

**Affiliations:** 1 Microbiology Department, Faculty of Science, Ain Shams University, Cairo, Egypt; 2 School of Biotechnology, Badr University in Cairo (BUC), Badr City, Cairo Egypt

**Keywords:** Thermophilic microorganisms, Hot springs, Biodegradation, Metagenomics, Bioremediation, Biological techniques, Biotechnology, Environmental sciences, Microbiology, Molecular biology

## Abstract

Thermophilic hot springs host highly specialized microbial communities critical for biogeochemical cycling and novel biotechnological applications. This study investigated the structure of the bacterial communities (bacteriomes) and predicted functional potential related to bioremediation and sulfur metabolism across three geochemically diverse soil sites within the Pharaoh’s Bath Hot Springs ecosystem in South Sinai, Egypt. These sites were categorized by distinct thermal profiles: 70 °C (HS1), 75 °C (HS2), and 80 °C (HS3). Using 16 S rRNA gene amplicon sequencing and PICRUSt functional prediction, sequence analysis via the EzBioCloud server revealed that the HS2 site harbored the highest evenness and overall microbial diversity. Taxonomically, the HS1 and HS3 sites were dominated by *Proteobacteria*; in contrast, the HS2 site exhibited a more diverse profile, characterized by a reduced *Proteobacteria* presence and a high abundance of *Rhodothermaeota*. Predictive functional profiling identified 13 genes associated with biodegradation pathways (e.g., catechol and xylene degradation), suggesting an intrinsic genetic capacity to degrade complex aromatics and halogenated compounds across these thermal gradients. Regarding sulfur metabolism, functional predictions indicated that the HS2 site possessed the highest potential for dissimilatory sulfate reduction. Meanwhile, the HS1 site specialized in assimilatory sulfate reduction and, alongside the HS2 site, demonstrated a higher predicted capacity for sulfide oxidation. The distribution of heat-response genes varied by location: HspQ and Hsp33 were most prominent at the HS1 site, while HSP20 and DnaK reached their maximum abundance at the HS2 site. Overall, this study demonstrates the substantial intrinsic bioremediation potential of the studied bacteriomes and provides a predictive framework for understanding microbial functional potential in this system, with future studies offering opportunities to refine in situ functional validation and application.

## Introduction

Natural hot springs are widely distributed in various locations in the world. Geothermal environments are extreme biological niches that support the life of a variety of microorganisms^1^. Geothermal springs contain diverse groups of microorganisms^2^. The microbial diversity in these geothermal environments is governed by physicochemical parameters, including water chemistry, geological origin, pressure and geothermal gradient^3,4^. Identification and classification of the extremophiles is critical, as their metabolic diversity and stable extremozymes offer significant potential for industrial and biotechnological applications^5^.

In geothermally active and hypersaline sites, such as the hot springs of the Sinai Peninsula, the scarcity of organic carbon often necessitates a reliance on lithotrophic pathways. Sulfur cycling represents a critical biogeochemical engine, where specialized microbial consortia, especially sulfur-oxidizing bacteria (SOB) and sulfate-reducing bacteria (SRB) form intricate metabolic networks^6,7^. These microbial consortia drive the transformation of sulfur compounds across diverse oxidation states to maintain ecosystem stability^8^. These microorganisms utilize sophisticated enzymatic systems, including the sulfur oxidation (Sox) complex and dissimilatory sulfite reductase (Dsr) pathways, to thrive under poly-extreme conditions^9^. Beyond ecological maintenance, these sulfur-metabolizing extremophiles possess robust potential for bioremediation, offering novel mechanisms for the detoxification of heavy metals and the degradation of xenobiotic compounds in harsh industrial effluents^10^. The bioremediation capabilities of microbes isolated from hot spring systems have been demonstrated previously, as shown by studies identifying bacterial isolates from South African hot springs capable of producing enzymes relevant to degradation of polyaromatic hydrocarbons and dye pollutants^11^, as well as bacterial isolates from Peruvian hot springs with biotechnological potential for the remediation of environments contaminated with oil, metals, and plastics^12^.

Microbial mediated bioremediation of a vast array of contaminants has been proved to be a clean and cost-effective alternative to conventional treatment approaches^13^. The Pharaoh’s Bath (Hammam Pharaon) ecosystem in Sinai is a rare poly-extreme habitat where temperatures reach up to 92 °C and hypersalinity converge with one of the world’s highest natural sulfur concentrations^7^. Recent research emphasizes this site as a “biodiversity hotspot” for thermophilic actinomycetes and other extremophiles with significant metabolic versatility^8,14^. This unique functional potential makes the site a critical reservoir for “extremozymes” capable of degrading persistent organic pollutants, such as hydrocarbons and synthetic polymers, under harsh thermal and saline conditions that typically inhibit standard biological treatments^10,15^. Furthermore, the site’s proximity to the Gulf of Suez industrial zone introduces potential anthropogenic hydrocarbons, providing a clear rationale for investigating the functional potential of resident communities in degrading persistent organic pollutants.

New-generation sequencing technologies emerged as a powerful tool in characterizing microbes in specific environments. Microbial ecology of several hot springs around the world has been studied using 16 S rRNA amplicon sequencing^16–18^. The metagenomic study has led to the cultivation-independent and comprehensive investigation of microbial and functional diversity of the ecological niches. The analysis of culture-independent metagenome sequence data is helpful in elucidating the association among taxonomic composition, their functioning, and environmental feature^19^. Metabolically diverse bacteriomes have been described in hot springs with very different physicochemical parameters^18,20^, and some of their thermostable enzymes have been characterized as valuable biocatalysts with potential use in biotechnology and industry^21^.

Comparative metagenomic analyses of analogous geothermal systems emphasize that temperature and sulfur availability are the primary determinants of bacteriome assembly and functional diversity. Recent studies on the Red Sea coast demonstrate that these habitats serve as evolutionary reservoirs for extremophilic genera like *Geobacillus* and *Thermus*, which possess the functional potential to degrade complex xenobiotics and precipitate heavy metals. Collectively, these findings underscore that sulfur-rich hot springs are not merely isolated geological features but are active “biorefineries” where metabolic flexibility allows for the remediation of persistent pollutants under conditions that would denature standard biological catalysts^7,10^.

Despite the recognized ecological significance of the Pharaoh’s Bath ecosystem, there remains a substantial knowledge gap regarding the direct correlation between microbial diversity and in situ bioremediation efficiency. While high-throughput sequencing has begun to unravel the complex taxonomic assembly of these thermal habitats, more studies are required to determine how high levels of phylogenetic diversity translate into specialized functional performance for pollutant degradation. The extreme selective pressures of high temperature and salinity likely foster unique specialist taxa whose roles in sulfur cycling and bioremediation are not yet fully understood through diversity metrics alone^7,10^. Consequently, further empirical research is essential to move beyond descriptive diversity analysis and establish the mechanistic links between community composition and the metabolic resilience required for scalable bioremediation in harsh industrial contexts.

The present study aimed to explore and characterize the bacterial communities (bacteriomes) of the Pharaoh’s Bath hot spring, South Sinai, Egypt, using 16 S rRNA amplicon sequencing. This approach helps identify pollutant-degrading populations in this hot spring and assess their potential for diverse bioremediation activities. Additionally, the study focused on identifying predicted functional biomarkers that could be utilized for bioremediation purposes as further evidence for potential diverse bioremediation processes. Particular attention was also given to detecting biomarkers linked to sulfur cycling and heat stress response, highlighting their ecological and functional significance within the hot spring environment. Overall, this research aims to reveal the Pharaoh’s Bath hot spring as a promising reservoir of microbial resources with substantial biotechnological and bioremediation potential.

## Materials and methods

### Sampling procedure and sampling site

Soil sampling was conducted at the Pharaoh’s Bath (Hammam Pharaon) hot springs (29°12′24.9″ N, 32°57′35.4″ E) in the South Sinai Governorate, Egypt. Three distinct sampling sites were selected based on a perpendicular thermal gradient emerging from the primary geothermal source to capture the transition in bacteriome structure across varying heat intensities. The selected three sites represent different geographic distributions along with distinct thermal profiles: 70 °C (HS1), 75 °C (HS2), and 80 °C (HS3). At each site, soil was collected in triplicate to ensure a representative metagenomic profile. Samples were obtained from the subsurface layer at a depth of 5 cm using a sterile spatula. Approximately 50 g of soil was collected per subsample, and the three subsamples from each site were pooled at the time of collection to form a single composite sample in sterile polypropylene containers prior to DNA extraction. To maintain biological integrity, the composite samples were immediately placed in a portable icebox at 4 °C for transport and subsequently stored at − 20 °C until genomic DNA extraction was performed.

Overall, soil samples were selected over water samples because sediment/soil matrices have been reported to harbor higher microbial diversity^22^ and function as long-term reservoirs of microbial communities compared to the overlying water column^23^.

### DNA extraction and PCR amplification of the V3-V4 region of bacterial 16 S rRNA genes

Total genomic DNA was directly extracted from soil collected at the three sampling sites using the Soil DNA Isolation Kit (Mo Bio Laboratories, Solana Beach, CA, USA) following the manufacturer’s protocol. The hypervariable V3–V4 region of the bacterial 16 S rRNA gene was then amplified by PCR using the universal primers 341 F (5′-CCTACGGGNGGCWGCAG-3′) and 805R (5′-GACTACHVGGGTATCTAATCC-3′)^24^. PCR amplification was carried out according to the Illumina 16 S rRNA Metagenomic Sequencing Library Preparation protocol at Macrogen (Daejeon, Korea). The thermal cycling program consisted of an initial denaturation at 95 °C for 3 min, followed by 25 cycles of denaturation at 95 °C for 30 s, annealing at 55 °C for 30 s, and extension at 72 °C for 30 s, with a final extension at 72 °C for 5 min.

### Illumina miseq sequencing and bioinformatic processing of 16s rRNA gene amplicons

The PCR amplicons (~ 450 bp) obtained from the previous step were sequenced using a paired-end (2 × 300 bp) chemistry on the Illumina MiSeq platform at Macrogen (Daejeon, Korea). The resulting raw sequence reads (FASTQ format) were processed and analyzed using the Microbiome Taxonomic Profiling (MTP) pipeline^25^ provided by EzBioCloud (https://www.ezbiocloud.net/contents/16smtp). Initially, Illumina paired-end reads were merged using the VSEARCH program^26^, which is available under either the BSD 2-clause license or the GNU General Public License version 3.0 (https://github.com/torognes/vsearch). PCR primer sequences were subsequently removed using in-house code, followed by quality filtering to eliminate low-quality reads, such as reads with lengths < 100 bp or > 2,000 bp, average quality scores < 25, or sequences not identified as 16 S rRNA genes. Taxonomic assignment was performed by comparing sequences against the EzBioCloud 16 S database (PKSSU4.0) using VSEARCH program^26^(https://github.com/torognes/vsearch), applying a 97% similarity threshold for species-level classification, with lower similarity cutoffs for higher taxonomic ranks. Chimeric sequences were identified among unmatched reads using the UCHIME algorithm^27^(v4.2.52, http://drive5.com/uchime). Subsequently, operational taxonomic units (OTUs) were defined using an open-reference clustering approach, in which sequences were first aligned against reference databases, and any remaining unmatched reads were clustered using the UCLUST algorithm^28^(https://drive5.com/usearch/manual/uclust_algo.html) at a 97% similarity threshold.

Alpha diversity metrics, including Good’s coverage, rarefaction curves, observed OTUs, Chao1, Simpson, Shannon, and the Abundance-based Coverage Estimator (ACE), were calculated using the EZBioCloud server. ACE and Chao1 are commonly used estimators of species richness, representing the total number of species present in a sample^29,30^. In contrast, the Shannon and Simpson indices are measures of species diversity that account for both richness and evenness, reflecting not only the number of species but also the relative abundance distribution among them^31^. Beta diversity was assessed at the genus level using principal coordinate analysis (PCoA) based on Bray–Curtis distance matrices, as implemented in the EZBioCloud server. Rarefaction curve analysis was performed using the MG-RAST server^32^(version v4.0.3, https://www.mg-rast.org/) under default parameters. A Venn diagram (at the genus level) was created using the InteractiVenn online tool (https://www.interactivenn.net/) to illustrate the shared and unique OTUs among the three sampling sites. The phylogenetic tree, based on the neighbor-joining method, was constructed using MEGA11 software^33^(version 11, https://www.megasoftware.net/).

### Functional profiling and identification of potential biomarkers related to bioremediation, sulfur cycling and heat shock response

Predictive functional biomarkers profiling was conducted using the Phylogenetic Investigation of Communities by Reconstruction of Unobserved States (PICRUSt) tool^34^ integrated within the EZBioCloud platform, in combination with the Kyoto Encyclopedia of Genes and Genomes (KEGG) database^35^. The PICRUSt analysis applied the Kruskal–Wallis H test to determine significantly different functional profiles, using a significance threshold of *P* < 0.05^36^. Heatmaps illustrating potential functional biomarkers associated with the sulfur cycle and heat shock response, as predicted by PICRUSt, were generated using the SRplot online platform (https://www.bioinformatics.com.cn/en).

## Results

### General characteristics of hot springs-bacteriomes

**Fig. 1:**
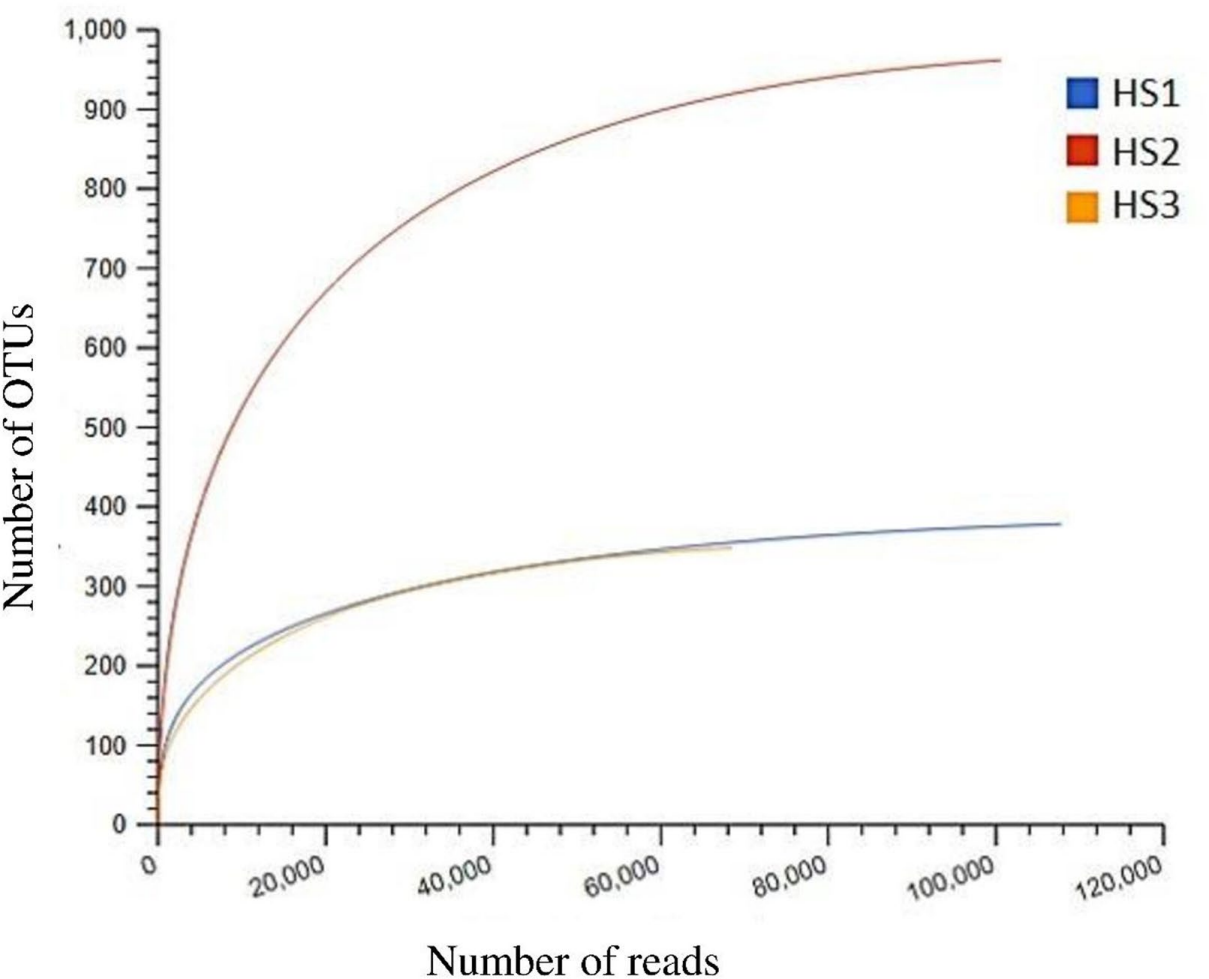
Rarefaction curves for the three bacteriomes identified in this study.

**Fig. 2:**
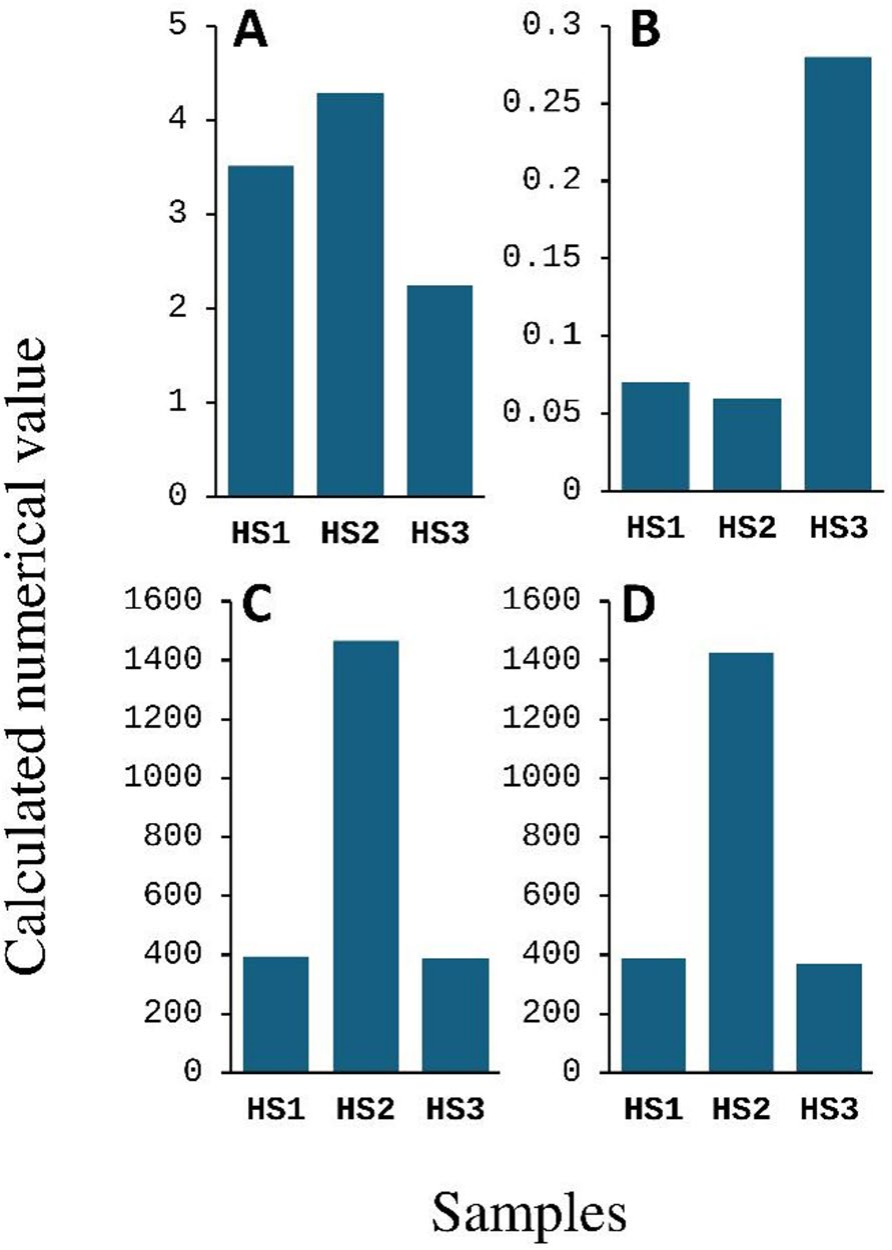
Alpha diversity indices illustrating bacterial richness and evenness across the identified bacteriomes: (**A**) Shannon, (**B**) Simpson, (**C**) ACE, and (**D**) Chao. For all indices, higher values indicate greater diversity, except for the Simpson index, where higher values correspond to lower diversity.

The present study employed 16 S rRNA MiSeq Illumina sequencing to investigate the bacteriome structure and composition in soil samples from Pharaoh’s Bath hot spring in South Sinai, Egypt. Analysis of raw 16 S rRNA gene sequences using the EZBioCloud server yielded 42,069 to 76,564 target reads and revealed 358 to 1,419 OTUs per sample (Table 1). Good’s coverage values exceeding 99% (Table 1) and plateaued rarefaction curves (Fig. 1) demonstrated sufficient sequencing depth for further analysis. The alpha diversity analysis, which measures diversity within the three identified bacteriomes (HS1, HS2, and HS3) using four indices, revealed distinct patterns of richness and evenness (Fig. 2). HS2 demonstrated the highest community evenness and overall diversity, scoring the highest on both the Shannon index and the Simpson Index. Conversely, HS3 exhibits the lowest values across all four metrics, indicating it is the least diverse, with the lowest estimated species richness and the lowest evenness. Notably, the richness-based estimators (ACE and Chao) show that HS1 and HS2 have very similar and high estimated total species numbers.

**Table 1:** Alpha diversity indices (valid reads, valid reads percentage, OTUs obtained and Good’s coverage of library (%)) identified in the three bacteriomes addressed in this study.

Sample name	Valid reads	Valid reads percentage	OTUs	Good’s coverage of library (%)
HS1	76,564	56.0%	373	99.95
HS2	55,050	63.4%	1419	99.82
HS3	42,069	59.8%	358	99.87

**Fig. 3:**
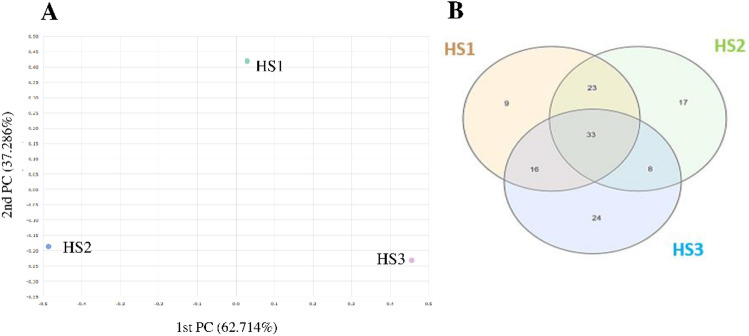
PCoA plot showing the clustering patterns of the three bacteriomes (**A**), and a Venn diagram illustrating the distribution of shared and unique OTUs among them (**B**).

PCoA plot, designed to visualize the differences in bacteriome structure, showed clear separation of the HS1, HS2, and HS3, indicating significant compositional differences between the three sampling sites (Fig. 3A). The greatest structural dissimilarity is observed between HS2 and HS3, indicating it has a unique bacteriome profile. Venn diagram analysis of genus-level composition (Fig. 3B) revealed that all three bacteriomes (HS1, HS2, and HS3) shared a substantial core bacteriome comprising 33 genera; however, the majority of the total 130 genera identified were unique. HS3 demonstrated the highest number of unique genera (24), followed by HS2 (17 unique genera), while HS1 had the fewest unique genera (9). Regarding pairwise overlap, HS1 and HS2 exhibited the strongest association, sharing 23 genera exclusively between them, whereas the overlap was weaker between HS1 and HS3 and weakest between HS2 and HS3, underscoring the compositional distinctiveness observed in the previous beta diversity analysis.

### Taxonomic affiliation of 16 S rRNA gene sequences

**Fig. 4:**
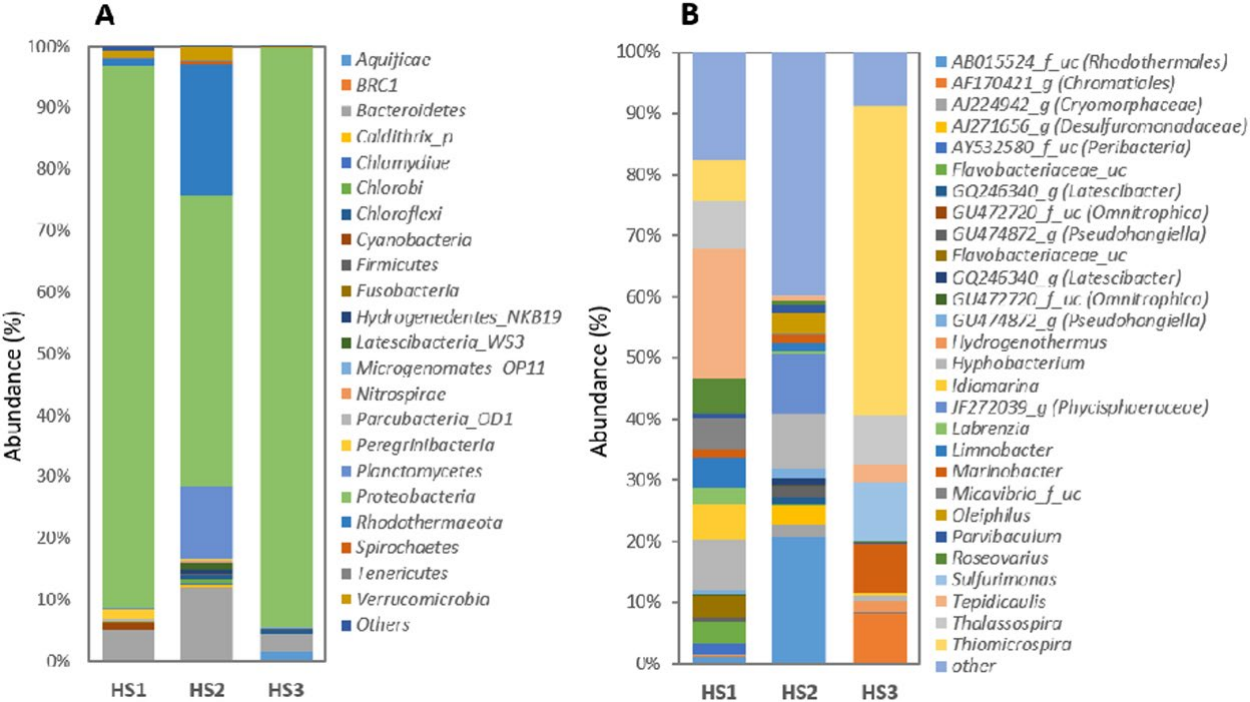
Bacterial community composition at the (**A**) phylum and (**B**) genus levels.

The analysis of microbial relative abundance at the phylum level (Fig. 4A) across the three groups reveals HS1 and HS3 are compositionally similar but distinctly different from HS2, due to the massive dominance of Proteobacteria, which constitutes approximately 88% of HS1 and nearly 94% of HS3. Bacteroidetes were the second most abundant phylum in HS1 (5.1%) and HS3 (2.743%). HS2 presents a far more diverse and complex profile, where the dominant Proteobacteria was significantly reduced to 46%. Rhodothermaeota dominated the second position in HS2 with 20.9% relative abundance. At the genus level (Fig. 4B), the relative abundance analysis showed a dramatic compositional variation between the three communities, with HS3 being exceptionally homogeneous, dominated by *Thiomicrospira*. HS1 was dominated by *Tepidicaulis* (21.29%) *Hyphobacterium* (8.36%) and Thalassospira (7.745%). HS2 shares dominance by AB015524_f_uc (from order Rhodothermales) (20.70%), JF272039_g (from family Phycisphaeraceae) (9.82%) and *Hyphobacterium* (9.02%). In addition to *Thiomicrospira* (50.58%), HS3 was co-dominated by *Sulfurimonas* (9.77%) and AF170421_g (from order Chromatiales) (8.0699%).

### Identification of genera with potential bioremediation capabilities

Based on previous reports, this study identified several bacterial genera with significant biodegradation potential (Table 2). These taxa include bacteria capable of degrading polycyclic aromatic hydrocarbons (PAHs) (e.g., *Pseudomonas*, *Acidovorax*), organohalide-respiring bacteria (OHRB) (e.g., *Pseudomonas* and *Desulfuromonas*), and metal-reducing bacteria (e.g., *Desulfovibrio*, *Shewanella*). Additionally, genera such as *Pseudomonas*, *Acinetobacter*, and *Alcanivorax* exhibited strong potential for oil and hydrocarbon degradation.

**Table 2:** Potential xenobiotic degrading genera identified in this study.

Genus	Pollutants	HS1	HS2	HS3	References
*Pseudomonas*	Crude oil, Polyethylene, Heavy oil, PAHs, OHRB	+	+	+	^37–41^
*Acidovorax*	PAHs, BTEX	+	-	-	^42,43^
*Acinetobacter*	Phenol, Petroleum Hydrocarbons	+	-	-	^44,45^
*Alcanivorax*	Beyond oil degradation, Long chain alkanes, Petroleum hydrocarbon	+	+	-	^46–48^
*Anoxybacillus*	Azo dye	+	-	-	^49^
*Arenibacter*	PAHs	-	+	-	^50^
*Bacillus*	Petroleum Hydrocarbon	-	+	+	^51^
*Defluviimonas*	PAHs	-	+	+	^52^
*Desulfovibrio*	U(VI) reduction	-	+	-	^53^
*Desulfuromonas*	OHRB, Fe (III), Mn, U (VI) reduction	+	+	-	^54–57^
*Marinobacter*	Hydrocarbons	+	+	+	^58^
*Nitrosomonas*	Quinoline, Crude oil	-	+	-	^59,60^
*Shewanella*	Iron, Mn, U (VI) reducing bacteria, Sulfonamides, PAHs	-	+	-	^54,61–63^
*Sulfurimonas*	Alkanes, PAHs, Nitrate-Reduction	-	-	+	^64,65^

### Functional biomarkers for bioremediation prediction

**Fig. 5:**
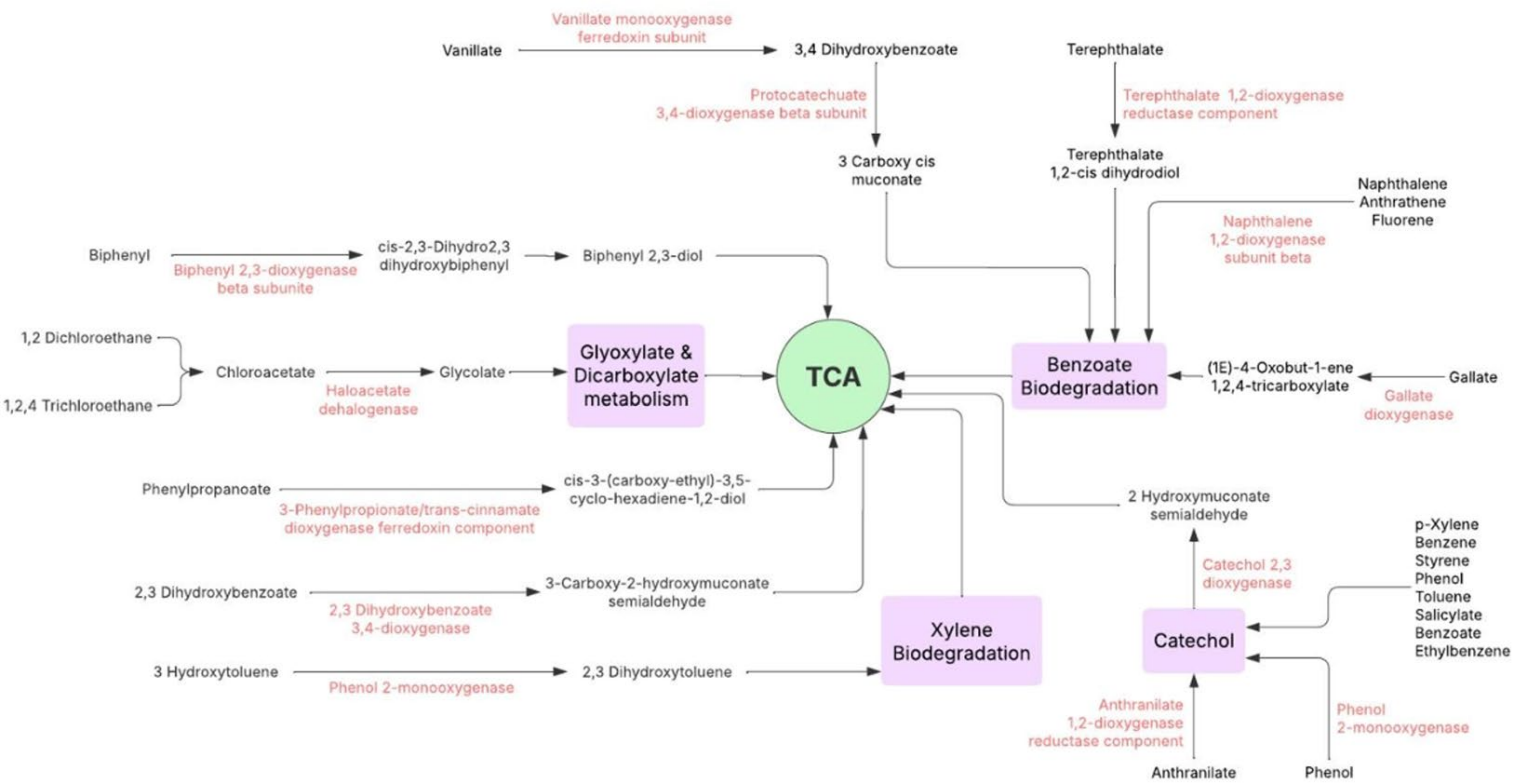
Xenobiotic degradation pathways proposed in this study. Functional biomarker genes predicted and detected using PICRUSt tool in this study are marked in red. Pathways were adapted from KEGG35. (permission number 254109).

PICRUSt analysis predicted the functional potential of the studied bacteriomes, indicating the presence of genes implicated in key enzymatic steps of biodegradation pathways. The red-labeled enzymes detailed in this metabolic map represent crucial functional biomarkers that directly relate to the bioremediation potential of the detected bacterial populations (Fig. 5). The functional analysis identified 13 highly significant predicted genes that serve as crucial functional biomarkers within the bacteriome. These genes are organized into four major catabolic pathways that ultimately feed into the TCA cycle. Specifically, the Catechol Bioremediation Pathway is characterized by genes for catechol 2,3-dioxygenase and phenol monooxygenase. The Benzoate Pathway involves genes encoding naphthalene dioxygenase and vanillate monooxygenase. The Glyoxylate & Dicarboxylate Metabolism pathway includes genes for haloacetate dehalogenase and biphenyl 2,3-dioxygenase. Finally, the Xylene Bioremediation Pathway is distinctively marked by the presence of the phenol monooxygenase gene. The provided metabolic map illustrates these pathways highlights a fundamental principle of microbial catabolism. Initial activation steps, catalyzed by key enzymes like Vanillate monooxygenase ferredoxin subunit, Biphenyl 2,3-dioxygenase beta subunit, and Naphthalene 1,2-dioxygenase subunit beta, facilitate the initial transformation of structurally diverse contaminants (e.g., biphenyl, naphthalene, vanillate, and chloroacetate). The products are then efficiently directed toward key central intermediates, primarily Catechol and Protocatechuate, catalyzed by enzymes such as Phenol 2-monooxygenase and Protocatechuate 3,4-dioxygenase subunit beta. This metabolic funnel ultimately directs chemical energy into the TCA cycle via the Benzoate Biodegradation and Glyoxylate & Dicarboxylate pathways. The identification of these predicted genes provides an indication of the potential metabolic capabilities of the bacteriomes based on taxonomic inference. Further studies may help to validate and refine the in situ activity and ecological relevance of these pathways, for example through shotgun metagenomics, transcriptomics, or cultivation-based approaches.

### Prediction of metabolic functions related to the sulfur cycle and heat stress response

**Fig. 6:**
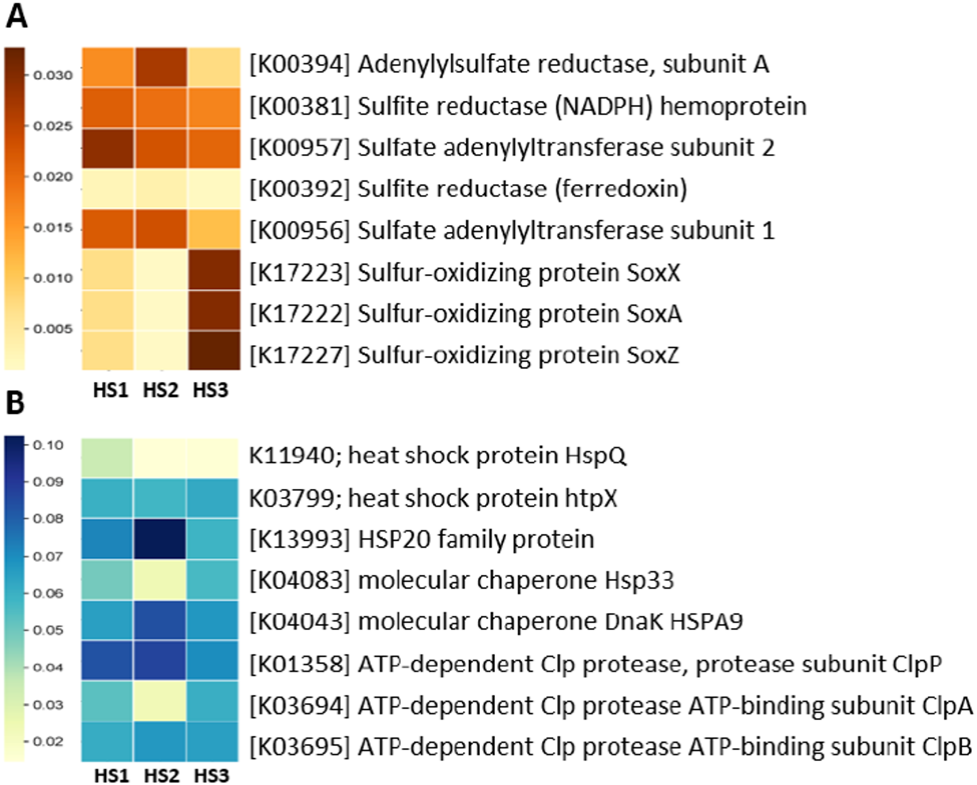
Heatmaps illustrating functional biomarkers predicted using PICRUSt: (**A**) sulfur cycle-related and (**B**) heat shock response-related biomarkers.

Due to the inherent sulfur-rich biogeochemistry of the sampling sites, particular emphasis was placed on analyzing functional biomarkers within the predicted bacteriome related to the sulfur cycle. This targeted investigation provides a direct link between the detected microbial taxa and the specific enzymatic machinery required to mediate the biogeochemical cycling of sulfur at the site, which is vital for understanding environmental health and potential remediation strategies. The heatmap analysis of PICRUSt-predicted genes for the sulfur cycle (Fig. 6A) reveals distinct specializations across the three communities. HS2 exhibits the highest genetic potential for dissimilatory sulfate reduction (DSR) (genes K00394 and K00392, involved in energy production), justifying its potential to thrive in anaerobic conditions by reducing sulfur compounds. Conversely, HS1 demonstrates the highest potential for assimilatory sulfate reduction (ASR) (genes K00381, K00957, K00956, necessary for biomass production), suggesting a focus on growth and sulfur incorporation into essential biomolecules. Furthermore, both HS1 and HS2 show a significantly higher genetic capacity for sulfide oxidation (SoxX, SoxA, and SoxZ genes) compared to HS3, justifying their ability to efficiently recycle reduced sulfur compounds and maintain sulfur redox homeostasis in their respective environments. In addition to the sulfur-rich characteristics of the sampling sites, special attention was given to identifying functional biomarkers and genes linked to the heat shock response considering the overall heat- and stress-associated nature of the sampling sites in this study. A broad range of heat-responsive genes was detected across the sampled sites. The distribution of these genes varied notably among the three sites (Fig. 6B). HspQ exhibited the highest abundance in HS1, followed by HS3 and HS2. Similarly, Hsp33 showed its highest level in HS1, with higher levels in HS3 than in HS2. Conversely, HSP20 is most represented in HS2, while DnaK is also highest in HS2. The protein htpX is most abundant in HS3. The protease, ClpP is most represented in HS2, while ClpA has its highest value in HS3, and ClpB is most abundant in HS2.

### Phylogenetic analysis and ecological interpretation

**Fig. 7:**
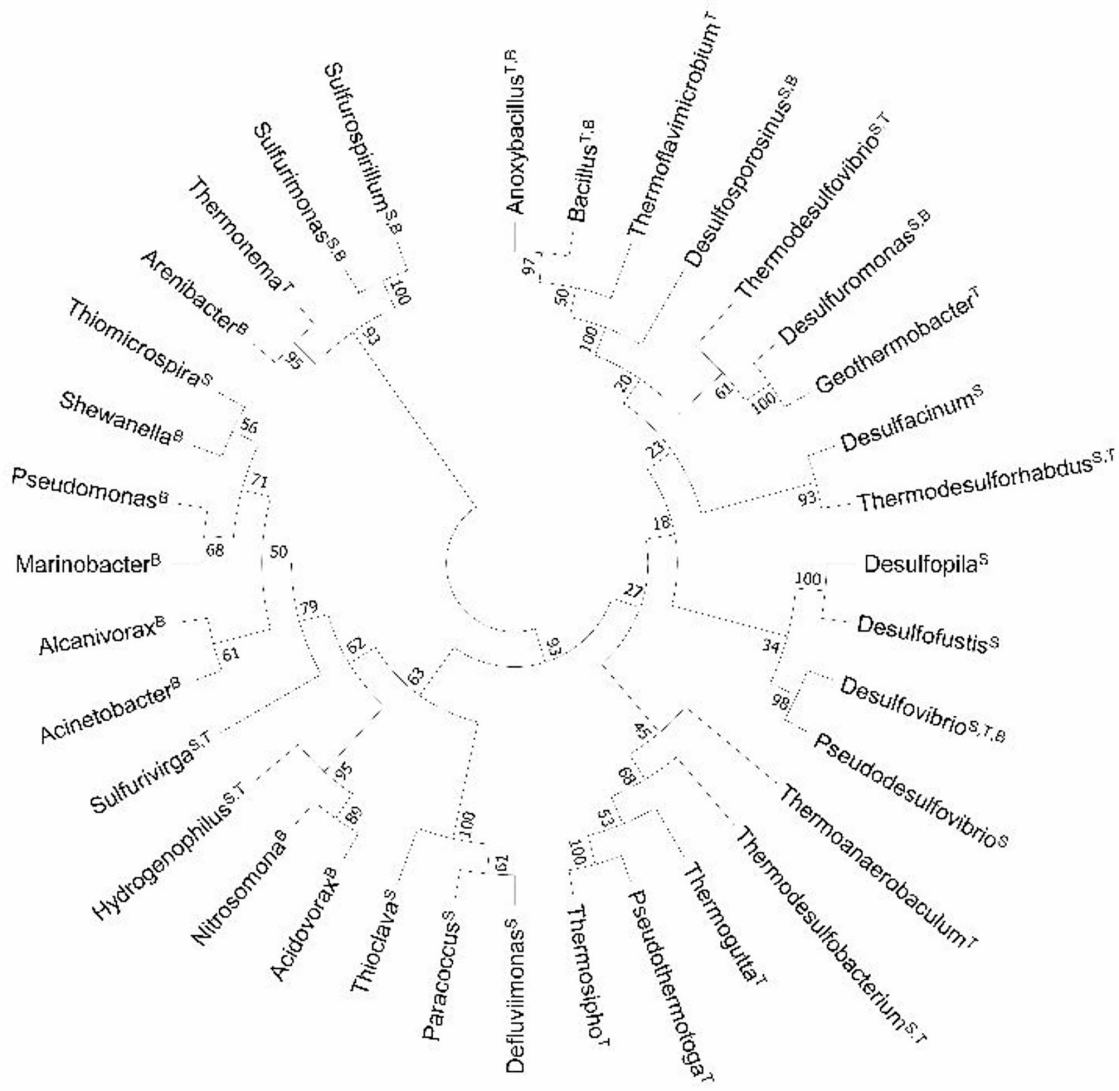
Neighbor-joining circular phylogenetic tree based on 16 S rRNA gene sequences, illustrating bacterial genera identified in this study that are potentially involved in xenobiotic biodegradation, sulfur cycling, and thermophily. Superscript letters following each genus indicate functional traits: (S) sulfur-metabolizing, (B) bioremediation-related, and (T) thermophilic genera. Multiple designations (e.g., S–B or S–T) reflect multifunctional roles in sulfur metabolism, thermophily, and bioremediation.

Figure (7) illustrates the phylogenetic relationships among the bacterial genera identified in this study that are potentially involved in xenobiotic degradation (Table 2), sulfur cycling, and thermophily (Table 3). based on a Neighbor-Joining tree constructed from 16 S rRNA gene sequences. The phylogenetic structure and functional profiling confirm that the analyzed samples harbor a diverse yet specialized community, optimized for biogeochemical sulfur cycling and adapted to thermophilic conditions, while simultaneously retaining high bioremediation potential. The tree is annotated to highlight three critical functional characteristics for each genus: (S) for sulfur metabolism (oxidation/reduction), (B) for bioremediation-related potential, and (T) for thermophilic adaptation. The distribution of functional characteristics across the tree suggests a community highly adapted to sulfur cycling and elevated temperatures, while also possessing significant potential for remediation. A large, strongly supported clade is characterized by genera possessing sulfur-metabolizing and thermophilic traits. This group includes several sulfate-reducing bacteria (SRB) like *Desulfovibrio*, *Thermodesulfovibrio*, and *Thermodesulforhabdus*, indicating that both sulfur reduction and oxidation are major processes in the analyzed samples. A separate, well-supported cluster comprises genera, primarily known for their roles in bioremediation. This includes genera like *Acinetobacter*, *Marinobacter*, and *Pseudomonas*, which are widely studied for their ability to degrade various organic pollutants. The presence of these genera suggests the bacteriome is resilient and capable of bioremediating diverse compounds. The most ecologically significant observation is the presence of several genera exhibiting overlapping functions. *Desulfovibrio* is uniquely categorized as S, T, and B, signifying its critical and versatile role as a high-temperature sulfate-reducer with potential applications in pollutant remediation. Similarly, *Sulfurimonas* (S, B) and *Sulfurospirillum* (S, B) couple sulfur cycling with bioremediation capabilities, highlighting the interconnected nature of biogeochemical processes within this microbial consortium.

**Table 3:** Potential sulfur-cycle–associated and thermophilic bacterial genera identified in this study.

Genus	HS1	HS2	HS3	References
Sulfur bacteria				
*Desulfacinum*	-	+	+	^66^
*Desulfofustis*	+	+	-	^67^
*Desulfopila*	-	+	-	^68^
*Desulfosporosinus*	-	+	-	^69^
*Desulfovibrio*	-	+	-	^70^
*Desulfuromonas*	-	+	-	^71^
*Thioalkalispira*	+	-	+	^72^
*Thioclava*	+	+		^73^
*Thermodesulforhabdus*	-	-	+	^74^
*Sulfurospirillum*	+	-	-	^75^
*Sulfurimonas*	-	-	+	^76^
*Sulfurivirga*	+	-	+	^77^
*Thiomicrospira*	+	-	+	^78^
*Paracoccus*	+	+	+	^79^
*Pseudodesulfovibrio*	+	+	-	^80^
Thermophilic bacteria				
*Anoxybacillus*	+	-	-	^81^
*Desulfacinum*	-	+	+	^82^
*Geothermobacter*	-	+		^83^
*Pseudothermotoga*	+	-	-	^84^
*Thermoanaerobaculum*	-	-	+	^85^
*Thermodesulfobacterium*	+	-	-	^86^
*Thermodesulforhabdus*	-	-	+	^87^
*Thermodesulfovibri*	-	+	-	^88^
*Thermoflavimicrobium*	-	+	-	^89^
*Thermogutta*	+	-	-	^90^
*Thermonema*	-	-	+	^91^
*Thermosipho*	+	-	+	^92^
*Hydrogenophilus*	+	-	+	^93^
*Sulfurivirga*	+	-	+	^94^

## Discussion

Pharaoh’s Bath (Hammam Pharaon), located about 250 km from Cairo, comprises natural sulfur-rich hot springs flowing into a 100-m lake by the sea and is well known for therapeutic tourism^95^. However, its microbiological profile remains largely unexplored. This study investigated the bacteriomes of three sites from these hot springs, focusing on genera associated with bioremediation and key functional biomarkers of these processes.

Thermally enhanced bioremediation is a novel concept that explores the combination of thermal treatment and bioremediation to enhance the low efficiency and long duration of bioremediation. The thermal effects on the physical, chemical and biological characteristics of soil, and contaminants bioavailability and bacterial commuity metabolic activities. Specifically, the increase in temperature within a suitable range can proliferate enzymes enrichment, extracellular polysaccharides and biosurfactants production, and further improving bioremediation efficacy^96^. Microorganisms that thrive at temperatures from 70 °C to 80 °C are classified as extreme thermophiles^97^. Moreover, a sufficient increase in temperature can enhance bioremediation. Yadav et al. (2012)^98^ recorded that the bioremediation of toluene was increased two folds for every 10 °C rise in soil temperature.

Proteobacteria dominated all samples, followed by Bacteroidetes in HS1 and HS3 and Rhodothermaeota in HS2. While this agrees with previous studies, the second most abundant phylum varied. Sharma et al. (2020)^99^ reported Proteobacteria and Firmicutes in Sikkim hot springs, and DeCastro et al. (2021)^100^ reported Proteobacteria and Deinococcus-Thermus in Spain. Similar dominance of Proteobacteria has been observed in the Himalayas^101^ and Kharasinpur^102^, whereas other sites showed different patterns, such as Actinobacteria in Rajgir, India^103^ and Firmicutes in Jizan, Saudi Arabia^104^.

The predominant genera varied across the three bacteriomes. HS1 was dominated by *Tepidicaulis*, *Hyphobacterium*, and *Thalassospira*; HS2 by AB015524_f_uc (Rhodothermales), JF272039_g (Phycisphaeraceae), and *Hyphobacterium*; and HS3 by *Thiomicrospira*, *Sulfurimonas*, and AF170421_g (Chromatiales). These findings align with previous studies highlighting hot spring microbial diversity, such as the abundance of *Exiguobacterium* and *Pseudomonas* in Polok and *Thermodesulfovibrio* and *Sulfurihydrogenibium* in MDV^89,90^. Similar variability has been reported in Pharaoh’s Bath, where different studies identified genera such as *Geobacillus*, *Rhodothermus*, *Thermus*^105^, *Bacillus*, *Anoxybacillus*, and *Geobacillus*^106^ using conventional and molecular methods.

16 S rRNA gene analysis of the three sites identified microbial taxa with potential degradative functions (Tables 2 and 3). Proteobacteria, commonly dominant in hydrocarbon-contaminated environments, plays a key role in biodegradation^107^, alongside Actinobacteria, due to their specialized degradative enzymes. The dominance of Proteobacteria in these samples therefore indicates a strong potential for hydrocarbon biodegradation.

Functional prediction using PICRUSt suggested 13 key genes linked to major biodegradation pathways, supporting a strong degradation potential in the studied bacteriomes. These suggested genes are associated with the breakdown of a wide range of pollutants, including aromatic hydrocarbons, phenols, halogenated compounds, pharmaceuticals, and plastic-derived substances^108–113^, supporting a strong biodegradation potential in the studied bacteriomes. Similar findings were reported by Saxena et al. (2017)^18^, who identified abundant hydrocarbon degradation genes in Anhoni hot springs, dominated by *Pseudomonas stutzeri* and *Acidovorax* sp. Additionally, Saeed et al. (2020)^114^ demonstrated molybdate bioremediation in Pharaoh’s Bath, further highlighting its environmental remediation potential of the studied site^115^.

In addition to hydrocarbon degradation, sulfur metabolism pathways were also predicted. PICRUSt analysis identified genes associated with both DSR and ASR pathways. In DSR, the aprA gene (K00394) involved in APS-to-sulfite conversion was detected, although additional genes are required for complete sulfate reduction^116,117^. In ASR, key genes including cysN (K00956), cysD (K00957), cysI (K00381), and sir (K00392) were identified, supporting partial pathway functionality. The presence of SOX genes further indicates thiosulfate oxidation potential, relevant for treating sulfur-rich industrial wastewater^118–121^. Similar sulfur metabolism genes have been reported in hot springs from Taiwan^122^ and New Zealand^123^. Together, these findings highlight an expanded bioremediation potential through sulfur cycling, complementing previously identified hydrocarbon degradation capabilities.

PICRUSt analysis also predicted a diverse set of heat shock protein genes, indicating strong microbial adaptation to environmental stress. These proteins support cellular stability by maintaining protein folding, preventing aggregation, and facilitating repair under extreme conditions. Key HSPs such as DnaK, ClpP, Hsp33, and HSP20 are associated with tolerance to thermal, oxidative, and chemical stress^124–129^, reflecting active stress response mechanisms within the bacteriomes.

Although Archaea, eukaryotes, and viruses occur in hot spring environments, numerous studies show that bacteria dominate both community abundance and sulfur-related functional potential in many systems^130,131^. In some surveys, bacteria account for up to 95% or more of total microbial sequences, while Archaea represent only a small fraction^132^. Eukaryotes are also minor contributors, comprising about 1.3% of taxa in hot spring biofilms. Viruses are present, but their ecological roles especially in sulfur cycling remain less well characterized^133^.

Bacteriome profiling in this study was conducted using 16 S rRNA gene amplicon sequencing, a widely applied and cost-effective approach for characterizing microbial diversity. Although shotgun metagenomics provides higher taxonomic resolution and direct functional insights, its greater cost and computational demands often limit its overall use^134^. While this method provides a rapid overview of community composition, it is inherently limited by primer bias, variation in gene copy number, and reduced taxonomic resolution at the species level^135,136^. Moreover, functional predictions inferred using PICRUSt are based on taxonomic profiles and should therefore be considered indicative rather than confirmatory.

Future work should focus on integrating high-resolution physicochemical characterization (e.g., salinity, pH, metal concentrations) with multi-omics approaches to strengthen ecological interpretation. Specifically, shotgun metagenomics targeting key functional genes involved in pollutants degradation will provide direct evidence of metabolic capacity. In addition, enrichment and isolation of key thermophilic taxa will enable functional validation under controlled laboratory conditions. Finally, in situ or pilot-scale bioreactor experiments using industrial effluents are recommended to evaluate the practical applicability and stability of these microbial communities under real-world conditions. Such approaches will be essential to confirm the functional potential inferred in this study and to bridge the gap between prediction and application.

## Conclusion

This study offers an overview of the taxonomic composition and inferred metabolic potential of thermophilic bacteriomes inhabiting Pharaoh’s Bath Hot Springs in South Sinai, Egypt. The results reveal distinct community structures among the three sites, likely shaped by temperature gradients and associated geochemical conditions, reflecting strong environmental selection pressures. Predictive functional analyses indicate the possible presence of genes putatively involved in sulfur cycling, stress response, and xenobiotic degradation. Collectively, the hot springs studied represent promising reservoirs of bacterial communities with potential biotechnological and bioremediation applications. These PICRUSt-based functional insights should be interpreted with caution, as they do not reflect direct measurements of gene expression or activity, but rather indicate potential trends. Therefore, validation using complementary approaches such as shotgun metagenomics, metatranscriptomics, and targeted assays is required. Future work should integrate physicochemical characterization with multi-omics analyses targeting key sulfur and biodegradation related genes, alongside enrichment of key taxa and bioreactor-based experiments to confirm functional potential under environmentally relevant and application-relevant conditions.

## Data Availability

Raw sequence data from this research have been submitted to the NCBI database and can be accessed via the BioProject accession number PRJNA1354486.

## References

[1] Roy, C. et al. Microbiome and ecology of a hot spring-microbialite system on the Trans-Himalayan Plateau. *Sci. Rep.* **10**, 5917 (2020). 32246033 10.1038/s41598-020-62797-z PMC7125080

[2] Satyanarayana, T., Raghukumar, C. & Shivaji, S. Extremophilic microbes: diversity and perspectives. *Curr. Sci.* **89** (1), 78–90 (2005).

[3] Rampelotto, P. H. Extremophiles and extreme environments. *Life* **3**, 482–485. 10.3390/life3030482 (2013). 25369817 10.3390/life3030482 PMC4187170

[4] Merino, N. et al. Giovannelli, D. Living at the extremes: extremophiles and the limits of life in a planetary context. *Front. Microbiol.* **10** 780–804 (2019). 10.3389/fmicb.2019.00780 PMC6476344 31037068

[5] Mehta, R., Singhal, P., Singh, H., Damle, D. & Sharma, A. K. Insight into thermophiles and their wide-spectrum applications. *3 Biotech.* **6**, 81 (2016). 28330151 10.1007/s13205-016-0368-z PMC4764608

[6] Osman, Y. A., Gbr, M. M., Abdelrazak, A. & Mowafy, A. M. Fatty acids and survival of bacteria in Hammam Pharaon springs, Egypt. *Egypt. J. Basic. Appl. Sci.* **5** (2), 169–175 (2018).

[7] Helmi, N. R. Exploring the diversity and antimicrobial potential of actinomycetes isolated from different environments in Saudi Arabia: a systematic review. *Front. Microbiol.* **16**, 1568899 (2025). 40207161 10.3389/fmicb.2025.1568899 PMC11979186

[8] Gupta, S., Plugge, C. M. & Muyzer, G. Sánchez-Andrea, I. Harnessing the potential of the microbial sulfur cycle for environmental biotechnology. *Curr. Opin. Biotechnol.* **88**, 103164 (2024). 38964081 10.1016/j.copbio.2024.103164

[9] Vavourakis, C. D. et al. A metagenomics roadmap to the uncultured genome diversity in hypersaline soda lake sediments. *Microbiome* **6** (1), 168 (2018). 30231921 10.1186/s40168-018-0548-7 PMC6146748

[10] Hashim, H. S., Zayan, M. M. & Mohamed, A. A. El rahman Abulila, H. I. A. Actinomycetes in the spotlight: biodiversity and their role in bioremediation. *World J. Microbiol. Biotechnol.* **42** (2), 44 (2026). 41545732 10.1007/s11274-025-04610-5 PMC12811286

[11] Jardine, J. L., Stoychev, S., Mavumengwana, V. & Ubomba-Jaswa, E. Screening of potential bioremediation enzymes from hot spring bacteria using conventional plate assays and liquid chromatography-Tandem mass spectrometry (Lc-Ms/Ms). *J. Environ. Manage.* **223**, 787–796 (2018). 29986326 10.1016/j.jenvman.2018.06.089

[12] Valdez-Nuñez, L. F. & Rivera-Jacinto, M. A. Thermophilic bacteria from Peruvian hot springs with high potential application in environmental biotechnology. *Environ. Technol.* **45** (7), 1420–1435 (2024). 36356186 10.1080/09593330.2022.2143293

[13] Sar, P., Kazy, S. K. & Paul, D. Sarkar A Metal bioremediation by thermophilic microorganisms. In: (eds Satyanarayana, T., Littlechild, J. & Kawarabayasi, Y.) Thermophilic microbes in environmental and industrial biotechnology. Springer, Dordrecht, 171–201 (2013).

[14] Kortam, Y. G. et al. Enhancing the antibiotic production by thermophilic bacteria isolated from hot spring waters via ethyl methanesulfonate mutagenesis. *Antibiotics* **12** (7), 1095 (2023). 37508191 10.3390/antibiotics12071095 PMC10376502

[15] Alsharif, W., Saad, M. M. & Hirt, H. Desert microbes for boosting sustainable agriculture in extreme environments. *Front. Microbiol.* **11**, 1666 (2020). 32793155 10.3389/fmicb.2020.01666 PMC7387410

[16] Wemheuer, B., Taube, R., Akyol, P., Wemheuer, F. & Daniel, R. Microbial diversity and biochemical potential encoded by thermal spring metagenomes derived from the Kamchatka Peninsula. *Archaea* **2013**, 136714 (2013). 23533327 10.1155/2013/136714 PMC3600328

[17] Verma, A., Gupta, M. & Shirkot, P. Isolation and characterization of thermophilic bacteria in natural hot water springs of Himachal Pradesh (India). *Bioscan* **9**, 947–952 (2014).

[18] Saxena, R. et al. Metagenomic analysis of hot springs in central India reveals hydrocarbon degrading thermophiles and pathways essential for survival in extreme environments. *Front. Microbiol.* **7**, 2123 (2017). 10.3389/fmicb.2016.02123 PMC5214690 28105025

[19] Bougnom, B. P. et al. Wastewater used for urban agriculture in West Africa as a reservoir for antibacterial resistance dissemination. *J. Environ. Res.* **168**, 14–24 (2019). 10.1016/j.envres.2018.09.022 30253312

[20] Sahay, H. et al. Hot springs of Indian Himalayas: potential sources of microbial diversity and thermostable hydrolytic enzymes. *3 Biotech.* **7**, 1–11 (2017). 10.1007/s13205-017-0762-1 PMC5451362 28567630

[21] Ferrandi, E. E. et al. New thermophilic α/β class epoxide hydrolases found in metagenomes from hot environments. *Front. Bioeng. Biotechnol.* **6**, 144 (2018). 30386778 10.3389/fbioe.2018.00144 PMC6198070

[22] Colman, D. R. et al. Ecological differentiation in planktonic and sediment-associated chemotrophic microbial populations in Yellowstone hot springs. *FEMS Microbiol. Ecol.* **92** (9), fiw137 (2016). 27306555 10.1093/femsec/fiw137

[23] Samson, R., Kumar, S., Dastager, S., Khairnar, K. & Dharne, M. Deciphering the comprehensive microbiome of glacier-fed Ganges and functional aspects: implications for one health. *Microbiol. Spectr.* **13** (8), e01720–e01724 (2025). 40621926 10.1128/spectrum.01720-24 PMC12323359

[24] Takahashi, S., Tomita, J., Nishioka, K., Hisada, T. & Nishijima, M. Development of a prokaryotic universal primer for simultaneous analysis of Bacteria and Archaea using next-generation sequencing. *PLoS ONE* **9**(8), e105592. (2014). **pmid:25144201**. 25144201 10.1371/journal.pone.0105592 PMC4140814

[25] Yoon, S. H. et al. Introducing EzBioCloud: a taxonomically united database of 16S rRNA gene sequences and whole-genome assemblies. *Int. J. Syst. Evol. Microbiol.* **67**, 1613 (2017). 28005526 10.1099/ijsem.0.001755 PMC5563544

[26] Rognes, T., Flouri, T., Nichols, B., Quince, C. & Mahe, F. VSEARCH: a versatile open source tool for metagenomics. *PeerJ* **4**, e2584 (2016). 27781170 10.7717/peerj.2584 PMC5075697

[27] Edgar, R. C., Haas, B. J., Clemente, J. C., Quince, C. & Knight, R. UCHIME improves sensitivity and speed of chimera detection. *Bioinformatics* **27**, 2194–2200 (2011). 21700674 10.1093/bioinformatics/btr381 PMC3150044

[28] Edgar, R. C. Search and clustering orders of magnitude faster than BLAST. *Bioinform* **26** (19), 2460–2461 (2010). 10.1093/bioinformatics/btq461 20709691

[29] Chao, A. & Lee, S. M. Estimating the number of classes via sample coverage. *J. Am. Stat. Assoc.* **87** (417), 210–217 (1992).

[30] Chao, A. Estimating the population size for capture-recapture data with unequal catchability. *Biometrics*, **43** 783–791 (1987). 3427163

[31] Magurran, A. E. *Measuring biological diversity* (Wiley, 2013). 10.1016/j.cub.2021.07.049 34637726

[32] Meyer, F. et al. The metagenomics RAST server–a public resource for the automatic phylogenetic and functional analysis of metagenomes. *BMC Bioinform.* **9** (1), 386 (2008). 10.1186/1471-2105-9-386 PMC2563014 18803844

[33] Tamura, K., Stecher, G. & Kumar, S. MEGA11: molecular evolutionary genetics analysis version 11. *Mol. Biol. Evol.* **38** (7), 3022–3027 (2021). 33892491 10.1093/molbev/msab120 PMC8233496

[34] Langille, M. G. et al. Predictive functional profiling of microbial communities using 16S rRNA marker gene sequences. *Nat. Biotechnol.* **31** (9), 814–821 (2013). 23975157 10.1038/nbt.2676 PMC3819121

[35] Kanehisa, M., Goto, S., Sato, Y., Furumichi, M. & Tanabe, M. KEGG for integration and interpretation of large-scale molecular data sets. *Nucleic Acids res.* **40** (D1), D109–114 (2012). 22080510 10.1093/nar/gkr988 PMC3245020

[36] Kim, S. S. et al. Microbiome as a potential diagnostic and predictive biomarker in severe alcoholic hepatitis. *Aliment. Pharmacol. Ther.* **53** (4), 540–551 (2021). 33264437 10.1111/apt.16200

[37] Ehmedan, S. S., Ibrahim, M. K., Azzam, A. M., Hamedo, H. A. & Saeed, A. M. Acceleration the bacterial biodegradation of crude oil pollution using Fe2O3 and ZnO nanoparticles. *Environ. Nanatechnol. Monit. Manage.* **16**, 100613 (2021).

[38] Hou, L. et al. Biodegradability of polyethylene mulching film by two Pseudomonas bacteria and their potential degradation mechanism. *Chemosphere* **286**, 131758 (2022). 34399255 10.1016/j.chemosphere.2021.131758

[39] Xia, W. et al. S-Heterocycles biodegradation and biosurfactant production under CO2/N2 conditions by Pseudomonas and its application on heavy oil recovery. *Chem. Eng. J.* **413**, 128771 (2021).

[40] Xia, W. et al. Biosurfactant produced by novel *Pseudomonas* sp. WJ6 with biodegradation of n-alkanes and polycyclic aromatic hydrocarbons. *J. Hazard. Mater.* **276**, 489–498 (2014). 24929788 10.1016/j.jhazmat.2014.05.062

[41] Chen, X. et al. How *Pseudomonas* conducts reductive dechlorination of 2, 4, 6-trichlorophenol: Insights into metabolic performance and organohalide respiration process. *Water Res.* **273**, 123014 (2025). 39719803 10.1016/j.watres.2024.123014

[42] Ramirez, D. R. & Aitken, L. G. Characterization of a polycyclic aromatic hydrocarbon degradation gene cluster in a phenanthrene-degrading *Acidovorax* strain. *Appl. Environ. Microbiol.* **75**, 2613–2620 (2009). 19270134 10.1128/AEM.01955-08 PMC2681696

[43] Benedek, T. et al. Aerobic and oxygen-limited enrichment of BTEX-degrading biofilm bacteria: dominance of Malikia versus *Acidovorax* species. *Environ. Sci. Pollut Res.* **25**, 32178–32195 (2018). 10.1007/s11356-018-3096-6 30220065

[44] Dankaka, S. M., Muhammad, J. B., Usman, S., Jagaba, A. H. & Abdullahi, N. Phenol biodegradation by Acinetobacter baumanii and Citrobacter sedlakii isolated from petroleum products contaminated environment. *Case Stud. Chem. Environ. Eng.* **8**, 100468 (2023).

[45] Cai, Y. et al. Bioremediation of petroleum hydrocarbons using Acinetobacter sp. SCYY-5 isolated from contaminated oil sludge: Strategy and effectiveness study. *Int. J. Environ. Res. Public Health*. **18** (2), 819 (2021). 33477988 10.3390/ijerph18020819 PMC7835959

[46] Zadjelovic, V. et al. Beyond oil degradation: enzymatic potential of *Alcanivorax* to degrade natural and synthetic polyesters. *Environ. Microbiol.* **22** (4), 1356–1369 (2020). 32079039 10.1111/1462-2920.14947 PMC7187450

[47] Wang, W. & Shao, Z. The long-chain alkane metabolism network of *Alcanivorax* dieselolei. *Nat. Commun.* **5** (1), 1–11 (2014). 10.1038/ncomms6755 25502912

[48] Kadri, T., Magdouli, S., Rouissi, T. & Brar, S. K. Ex-situ biodegradation of petroleum hydrocarbons using *Alcanivorax borkumensis* enzymes. *Biochem. Eng. J.* **132**, 279–287 (2018).

[49] Chen, G. et al. Detoxification of azo dye Direct Black G by thermophilic *Anoxybacillus* sp. PDR2 and its application potential in bioremediation. *Ecotoxicol. Environ. Saf.* **214**, 112084 (2021). 33640726 10.1016/j.ecoenv.2021.112084

[50] Gutierrez, T. et al. Polycyclic aromatic hydrocarbon degradation of phytoplankton-associatedArenibacter spp. and description of Arenibacter algicola sp. nov., an aromatic hydrocarbon-degrading bacterium. *Appl. Environ. Microbiol.* **80** (2), 618–628 (2014). 24212584 10.1128/AEM.03104-13 PMC3911095

[51] Baburam, C., Mitema, A., Tsekoa, T. & Feto, N. A. *Bacillus* species and their invaluable roles in petroleum hydrocarbon bioremediation. (eds Islam, M.T., Rahman, M., Pandey, P) In Bacilli in Agrobiotechnology: Plant Stress Tolerance, Bioremediation, and Bioprospecting (101–126). Cham: Springer International Publishing (2022).

[52] Zhang, S. et al. Defluviim*onas pyrenivorans* sp. nov., a novel bacterium capable of degrading polycyclic aromatic hydrocarbons. *Int. J. Syst. Evol. Microbiol.* **68** (3), 957–961 (2018). 29458487 10.1099/ijsem.0.002629

[53] You, W., Peng, W., Tian, Z. & Zheng, M. Uranium bioremediation with U (VI)-reducing bacteria. *Sci. Total Environ.* **798**, 149107 (2021). 34325147 10.1016/j.scitotenv.2021.149107

[54] Adrian, L. & Löffler, F. E. (eds) *Organohalide-respiring bacteria* Vol. 85 (Springer, 2016).

[55] Löffler, F. E., Sun, Q., Li, J. & Tiedje, J. M. 16S rRNA gene-based detection of tetrachloroethene-dechlorinating Desulfuromonas and Dehalococcoides species. *Appl. Environ. Microbiol.* **66** (4), 1369–1374 (2000). 10742213 10.1128/aem.66.4.1369-1374.2000 PMC91994

[56] Atashgahi, S., Lu, Y. & Smidt, H. Overview of known organohalide-respiring bacteria—phylogenetic diversity and environmental distribution. (eds Adrian, L., Löffl er, F.) In Organohalide-respiring bacteria (63–105). Springer, Berlin, Heidelberg (2016).

[57] Roden, E. E. & Lovley, D. R. Dissimilatory Fe (III) reduction by the marine microorganism *Desulfuromonas acetoxidans*. *Appl. Environ. Microbiol.* **59** (3), 734–742 (1993). 16348888 10.1128/aem.59.3.734-742.1993 PMC202183

[58] Chernikova, T. N. et al. Hydrocarbon-Degrading Bacteria Alcanivorax and Marinobacter Associated With Microalgae Pavlova lutheri and Nannochloropsis oculata. *Front. Microbiol.* **28** (11), 572931 (2020). 10.3389/fmicb.2020.572931 PMC7655873 33193176

[59] Chawley, P. & Jagadevan, S. Biodegradation of quinoline by *Nitrosomonas mobilis* Ms1 through nitrification: A mechanistic study. *Biochem. Eng. J.* **196**, 108933 (2023).

[60] John, R. C. & Okpokwasili, G. C. Crude oil-degradation and plasmid profile of nitrifying bacteria isolated from oil-impacted mangrove sediment in the Niger Delta of Nigeria. *Bull. Environ. Contam. Toxicol.* **88** (6), 1020–1026 (2012). 22460804 10.1007/s00128-012-0609-8 PMC3339056

[61] Roh, Y. et al. Metal reduction and iron biomineralization by a psychrotolerant Fe-(III) reducing bacterium, Shewanella sp. strain PV-4. *Appl. Environ. Microbiol.* **72** (5), 3236–3244 (2006). 16672462 10.1128/AEM.72.5.3236-3244.2006 PMC1472395

[62] Mao, F., Liu, X., Wu, K., Zhou, C. & Si, Y. Biodegradation of sulfonamides by *Shewanella oneidensis* MR-1 and *Shewanella* sp. strain MR-4. *Biodegradation* **29** (2), 129–140 (2018). 29302823 10.1007/s10532-017-9818-5

[63] Rathour, R., Medhi, K., Gupta, J. & Thakur, I. S. Integrated approach of whole-genome analysis, toxicological evaluation and life cycle assessment for pyrene biodegradation by a psychrophilic strain, *Shewanella* sp. *ISTPL2 Environ. Pollution*. **269**, 116176 (2021). 10.1016/j.envpol.2020.116176 33307397

[64] Wang, W., Li, Z., Zeng, L., Dong, C. & Shao, Z. The oxidation of hydrocarbons by diverse heterotrophic and mixotrophic bacteria that inhabit deep-sea hydrothermal ecosystems. *ISME J.* **14** (8), 1994–2006 (2020). 32355200 10.1038/s41396-020-0662-y PMC7368058

[65] Lahme, S. et al. Metabolites of an oil field sulfide-oxidizing, nitrate-reducing *Sulfurimonas* sp. cause severe corrosion. *Appl. Environ. Microbiol.* **85** (3), e01891–e01818 (2019). 30446554 10.1128/AEM.01891-18 PMC6344618

[66] Rozanova, E. P. et al. Desulfacinum subterraneumsp. Nov., a new thermophilic sulfate-reducing bacterium isolated from a high-temperature oil field. *Microbiology* **70** (4), 466–471 (2001). 11558281

[67] Watanabe, M., Takahashi, A., Kojima, H., Miyata, N. & Fukui, M. Desulfofustis *limnaeus* sp. nov., a freshwater sulfate-reducing bacterium isolated from marsh soil. *Arch. Microbiol.* **204** (10), 647 (2022). 36166176 10.1007/s00203-022-03261-6

[68] Gittel, A. et al. *Desulfopila inferna* sp. nov., a sulfate-reducing bacterium isolated from the subsurface of a tidal sand-flat. *Int. J. Syst. Evol. MicroBiol.* **60** (7), 1626–1630 (2010). 19717583 10.1099/ijs.0.015644-0

[69] Sánchez-Andrea, I., Stams, A. J., Hedrich, S., Ňancucheo, I. & Johnson, D. B. *Desulfosporosinus acididurans* sp. nov.: an acidophilic sulfate-reducing bacterium isolated from acidic sediments. *Extremophiles* **19** (1), 39–47 (2015). 25370366 10.1007/s00792-014-0701-6

[70] Fauque, G. et al. The three classes of hydrogenases from sulfate-reducing bacteria of the genus Desulfovibrio. *FEMS Microbiol. Rev.* **4** (4), 299–344 (1988). 3078655 10.1111/j.1574-6968.1988.tb02748.x

[71] Finster, K., Coates, J. D., Liesack, W. & Pfennig, N. *Desulfuromonas thiophila* sp. nov., a new obligately sulfur-reducing bacterium from anoxic freshwater sediment. *Int. J. Syst. Evol. MicroBiol.* **47** (3), 754–758 (1997). 10.1099/00207713-47-3-754 9226908

[72] Sorokin, D. Y., Tourova, T. Y. P., Kolganova, T. Y. V., Sjollema, K. A. & Kuenen, J. G. *Thioalkalispira microaerophila* gen. nov., sp. nov., a novel lithoautotrophic, sulfur-oxidizing bacterium from a soda lake. *Int. J. Syst. Evol. MicroBiol.* **52** (6), 2175–2182 (2002). 12508886 10.1099/00207713-52-6-2175

[73] Sorokin, D. Y., Tourova, T. P., Spiridonova, E. M., Rainey, F. A. & Muyzer, G. *Thioclava pacifica* gen. nov., sp. nov., a novel facultatively autotrophic, marine, sulfur-oxidizing bacterium from a near-shore sulfidic hydrothermal area. *Int. J. Syst. Evol. MicroBiol.* **55** (3), 1069–1075 (2005). 15879235 10.1099/ijs.0.63415-0

[74] Beeder, J., Torsvik, T. & Lien, T. *Thermodesulforhabdus norvegicus* gen. nov., sp. nov., a novel thermophilic sulfate-reducing bacterium from oil field water. *Arch. Microbiol.* **164** (5), 331–336 (1995). 8572886

[75] Finster, K., Liesack, W. & Tindall, B. J. *Sulfurospirillum arcachonense* sp. nov., a new microaerophilic sulfur-reducing bacterium. *Int. J. Syst. Evol. MicroBiol.* **47** (4), 1212–1217 (1997). 10.1099/00207713-47-4-1212 9336931

[76] Wang, S., Jiang, L., Liu, X., Yang, S. & Shao, Z. Sulfurimonas xiamenensis sp. nov. and Sulfurimonas lithotrophica sp. nov., hydrogen-and sulfur-oxidizing chemolithoautotrophs within the Epsilonproteobacteria isolated from coastal sediments, and an emended description of the genus Sulfurimonas. *Int. J. Syst. Evol. MicroBiol.* **70** (4), 2657–2663 (2020). 32134372 10.1099/ijsem.0.004087

[77] Takai, K. et al. Sulfurivirga caldicuralii gen. nov., sp. nov., a novel microaerobic, thermophilic, thiosulfate-oxidizing chemolithoautotroph, isolated from a shallow marine hydrothermal system occurring in a coral reef, Japan. *Int. J. Syst. evolutionary Microbiol.* **56** (8), 1921–1929 (2006). 10.1099/ijs.0.64297-0 16902032

[78] Sorokin, D. Y. et al. *Thiomicrospira halophila* sp. nov., a moderately halophilic, obligately chemolithoautotrophic, sulfur-oxidizing bacterium from hypersaline lakes. *Int. J. Syst. Evol. MicroBiol.* **56** (10), 2375–2380 (2006). 17012565 10.1099/ijs.0.64445-0

[79] Ghosh, W., Mandal, S. & Roy, P. *Paracoccus bengalensis* sp. nov., a novel sulfur-oxidizing chemolithoautotroph from the rhizospheric soil of an Indian tropical leguminous plant. *Syst. Appl. Microbiol.* **29** (5), 396–403 (2006). 16824961 10.1016/j.syapm.2005.10.004

[80] Frolova, A. A., Merkel, A. Y., Kuchierskaya, A. A., Bonch-Osmolovskaya, E. A. & Slobodkin, A. I. *Pseudodesulfovibrio alkaliphilus*, sp. nov., an alkaliphilic sulfate-reducing bacterium isolated from a terrestrial mud volcano. *Antonie van Leeuwenhoek*. **114** (9), 1387–1397 (2021). 34212258 10.1007/s10482-021-01608-5

[81] da Rosa, D. F. & Macedo, A. J. The genus *Anoxybacillus*: an emerging and versatile source of valuable biotechnological products. *Extremophiles* **16** (3), 22. 10.1007/s00792-023-01305-3 (2023). 10.1007/s00792-023-01305-3 37584877

[82] Rees, G. N., Grassia, G. S., Sheehy, A. J., Dwivedi, P. P. & Patel, B. K. *Desulfacinum infernum* gen. nov., sp. nov., a thermophilic sulfate-reducing bacterium from a petroleum reservoir. *Int. J. Syst. Evol. MicroBiol.* **45** (1), 85–89 (1995).

[83] Kashefi, K., Holmes, D. E., Baross, J. A. & Lovley, D. R. Thermophily in the Geobacteraceae: *Geothermobacter ehrlichii* gen. nov., sp. nov., a novel thermophilic member of the Geobacteraceae from the Bag City hydrothermal vent. *Appl. Environ. Microbiol.* **69** (5), 2985–2993 (2003). 12732575 10.1128/AEM.69.5.2985-2993.2003 PMC154550

[84] Farrell, A., Nesbø, C. L., Zhaxybayeva, O. & L’Haridon, S. *Pseudothermotoga*. *Bergey’s Man. Syst. Archaea Bacteria*, 1–12 (2015).

[85] McClure, P. J. Spore-forming bacteria (2006).

[86] Sonne-Hansen, J. & Ahring, B. K. *Thermodesulfobacterium hveragerdense* sp. nov., and *Thermodesulfovibrio islandicus* sp. nov., two thermophilic sulfate reducing bacteria isolated from a Icelandic hot spring. *Syst. Appl. Microbiol.* **22** (4), 559–564 (1999). 10794144 10.1016/S0723-2020(99)80009-5

[87] Galushko, A. & Kuever, J. *Thermodesulforhabdus*. Bergey’s Manual of Systematics of Archaea and Bacteria, 1–4 (2015).

[88] Maltseva, A. I. et al. *Thermodesulfovibrio autotrophicus* sp. nov., the first autotrophic representative of the widespread sulfate-reducing genus *Thermodesulfovibrio*, and *Thermodesulfovibrio obliviosus* sp. nov. that has lost this ability. *Syst. Appl. Microbiol.* **65** (3), 1490–1497 (2024). 10.1016/j.syapm.2024.126561 39551005

[89] Li, D. N., Huang, W. & Qiu, S. Y. *Thermoflavimicrobium daqui* sp. nov., a thermophilic microbe isolated from Moutai-flavour Daqu. *Int. J. Syst. Evol. MicroBiol.* **69** (9), 2709–2716 (2019). 31310191 10.1099/ijsem.0.003528

[90] Elcheninov, A. G. et al. Sugar Metabolism of the First Thermophilic Planctomycete *Thermogutta terrifontis*: Comparative Genomic and Transcriptomic Approaches. *Front. Microbiol.* **8**, 2140 (2017). 29163426 10.3389/fmicb.2017.02140 PMC5673643

[91] Hudson, J. A., Schofield, K. M., Morgan, H. W. & Daniel, R. M. *Thermonema lapsum* gen. nov., sp. nov., a thermophilic gliding bacterium. *Int. J. Syst. Evol. MicroBiol.* **39** (4), 485–487 (1989).

[92] Antoine, E. et al. *Thermosipho melanesiensis* sp. nov., a new thermophilic anaerobic bacterium belonging to the order Thermotogales, isolated from deep-sea hydrothermal vents in the southwestern Pacific Ocean. *Int. J. Syst. Bacteriol.* **47** (4), 1118–1123 (1997). 9336917 10.1099/00207713-47-4-1118

[93] Vesteinsdottir, H., Reynisdóttir, D. B. & Örlygsson, J. *Hydrogenophilus islandicus* sp. nov., a thermophilic hydrogen-oxidizing bacterium isolated from an Icelandic hot spring. *Int. J. Syst. Evol. MicroBiol.* **61** (2), 290–294 (2011). 20228213 10.1099/ijs.0.023572-0

[94] Takai, K. et al. Sulfurivirga caldicuralii gen. nov., sp. nov., a novel microaerobic, thermophilic, thiosulfate-oxidizing chemolithoautotroph,isolated from a shallow marine hydrothermal system occurring in a coral reef, Japan. *Int. J. Syst. Evol. MicroBiol.* **56** (8), 1921–1929 (2006). 16902032 10.1099/ijs.0.64297-0

[95] Shawky, A., El-Anbaawy, M. I., Shallaly, N. A. & Abdelhafiz, H. E. Geotouristic Potentiality Of Hammam Faraun Geothermal Area, South Sinai Governorate, Egypt. *Egypt. J. Geol.* **64**, 189–204 (2020).

[96] Wang, Q. et al. Thermally enhanced bioremediation: A review of the fundamentals and applications in soil and groundwater remediation. *J. Hazard. Mater.* **433**, 128749 (2022). 35364527 10.1016/j.jhazmat.2022.128749

[97] Li, J., Sun, L. & Huo, Y. X. High-temperature catalytic platform powered by thermophilic microorganisms and thermozymes. *Synth. Biology Eng.* **3** (1), 10001 (2025).

[98] Yadav, B. K., Shrestha, S. R. & Hassanizadeh, S. M. Biodegradation of toluene under seasonal and diurnal fluctuations of soil-water temperature. *Water Air Soil Pollut.* **223** (7), 3579–3588 (2012). 22865939 10.1007/s11270-011-1052-x PMC3409364

[99] Sharma, N. et al. Metagenomics revealing molecular profiling of community structure and metabolic pathways in natural hot springs of the Sikkim Himalaya. *BMC Microbiol.* **20** (1), 246 (2020). 32778049 10.1186/s12866-020-01923-3 PMC7418396

[100] DeCastro, M. E., Escuder-Rodríguez, J. J., Becerra, M., Rodríguez-Belmonte, E. & González-Siso, M. I. Comparative metagenomic analysis of two hot springs from Ourense (northwestern Spain) and others worldwide. *Front. Microbiol.* **12**, 769065 (2021). 34899652 10.3389/fmicb.2021.769065 PMC8661477

[101] Ishaque, K. et al. Amplicon Barcoding Based Sequencing Reveals Bacterial Diversity From Himalayan Hot spring of Kotli Azad Jammu and Kashmir (2024).

[102] Goswami, R., Sarkar, A., Bandyopadhyay, B. & Sadhukhan, S. Exploring microbial diversity in the Kharasinpur hot spring of West Bengal, India. *Mol. Biol. Rep.* **52** (1), 608 (2025). 40528093 10.1007/s11033-025-10690-1

[103] Kumari, A. & Rao, K. V. B. Exploring the Bacterial Diversity of Rajgir Hot Spring in India and its Antibacterial Potential. *Indian J. Microbiol.*, 1–8 (2025). 10.1007/s12088-025-01482-z PMC12579077 41180871

[104] AlSediy, K., Ashy, R., Al-fassi, F. & Al-Judaibi, A. Microbial diversity and abundance in the hot springs on the west coast of Saudi Arabia as a potential source of novel industrial products. *Microb. Biosystems J.* **7** (1), 8–17 (2022).

[105] Osman, Y., Mowafy, A., Abdelrazak, A. & El-Mallah, A. Identification of four thermophilic *Geobacillus* isolates from hammam pharaon, Sinai. *Egypt. J. Agric. Chem. Biotechn*. **9** (7), 151–157 (2018).

[106] Selim, S., Sherif, M. E., El-Alfy, S. & Hagagy, N. Genetic diversity among thermophilic bacteria isolated from geothermal sites by using two PCR typing methods. *Geomicrobiol J.* **31** (2), 161–170 (2014).

[107] Rahmeh, R. et al. N. Insights into bacterial community involved in bioremediation of aged oil-contaminated soil in arid environment. *Evolutionary Bioinf.* **17**, 11769343211016887 (2021). 10.1177/11769343211016887 PMC8191072 34163126

[108] Yu, C. L. et al. Purification, characterization, and crystallization of the components of a biphenyl dioxygenase system from *Sphingobium yanoikuyae* B1. *J. Ind. Microbiol. Biotechnol.* **34** (4), 311–324 (2007). 17211635 10.1007/s10295-006-0199-8

[109] Barber, E. A., Liu, Z. & Smith, S. R. Organic contaminant biodegradation by oxidoreductase enzymes in wastewater treatment. *Microorganisms* **8** (1), 122 (2020). 31963268 10.3390/microorganisms8010122 PMC7022594

[110] Mohapatra, B. & Phale, P. S. Microbial degradation of naphthalene and substituted naphthalenes: metabolic diversity and genomic insight for bioremediation. *Front. Bioeng. Biotechnol.* **9**, 602445 (2021). 33791281 10.3389/fbioe.2021.602445 PMC8006333

[111] Mesarch, M. B., Nakatsu, C. H. & Nies, L. Development of catechol 2, 3-dioxygenase-specific primers for monitoring bioremediation by competitive quantitative PCR. *Appl. Environ. Microbiol.* **66** (2), 678–683 (2000). 10653735 10.1128/aem.66.2.678-683.2000 PMC91880

[112] Silva, C. C. et al. Identification of genes and pathways related to phenol degradation in metagenomic libraries from petroleum refinery wastewater. *PloS one*. **8** (4), e61811 (2013). 23637911 10.1371/journal.pone.0061811 PMC3630121

[113] Guzik, U., Hupert-Kocurek, K., Sitnik, M., Wojcieszyńska, D. & Protocatechuate 3, 4-dioxygenase: a wide substrate specificity enzyme isolated from Stenotrophomonas maltophilia KB2 as a useful tool in aromatic acid biodegradation. *Microb. Physiol.* **24** (3), 150–160 (2014). 10.1159/000362791 24970342

[114] Saeed, A. M., Sayed, H. A. & El-Shatoury, E. H. Optimizing the reduction of molybdate by two novel thermophilic bacilli isolated from Sinai. *Egypt. Curr. Microbiol.* **77**, 786–794 (2020). 31925514 10.1007/s00284-020-01874-y

[115] Saeed, A. M., El-Shatoury, E. H. & Sayed, H. A. Statistical factorial designs for optimum production of thermostable α-amylase by the degradative bacterium *Parageobacillus thermoglucosidasius* Pharon1 isolated from Sinai, Egypt. *J. Genetic Eng. Biotechnol.* **19** (24), 1–9 (2021). 10.1186/s43141-021-00123-4 PMC7851196 33523315

[116] Li, J. et al. Bacterial community structure and predicted function in an acidogenic sulfate-reducing reactor: Effect of organic carbon to sulfate ratios. *Bioresour. Technol.* **293**, 122020 (2019). 31470231 10.1016/j.biortech.2019.122020

[117] Jin, Z., Liang, L., Zhao, Z. & Zhang, Y. Enhancing assimilatory sulfate reduction with ferrihydrite-humic acid coprecipitate in anaerobic sulfate-containing wastewater treatment. *Bioresour. Technol.* **411**, 131308 (2024). 39155018 10.1016/j.biortech.2024.131308

[118] Baquerizo, G., Chaneac, A., Arellano-García, L., González-Sánchez, A. & Revah, S. Biological removal of high loads of thiosulfate using a trickling filter under alkaline conditions. *Mine Water Environ.* **32** (4), 278–284 (2013).

[119] Gholipour, S. et al. Biological treatment of toxic refinery spent sulfidic caustic at low dilution by sulfur-oxidizing fungi. *J. Environ. Chem. Eng.* **6** (2), 2762–2767 (2018).

[120] Mohebali, G. & Ball, A. S. Biodesulfurization of diesel fuels – past, present and future perspectives. *Int. Biodeterior. Biodegrad*. **110** (Suppl. C), 163–180 (2016).

[121] Kalantari, H., Nosrati, M., Shojaosadati, S. A. & Shavandi, M. Investigation of transient forms of sulfur during biological treatment of spent caustic. *Environ. Technol.* **39** (12), 1597–1606 (2018). 28554258 10.1080/09593330.2017.1334707

[122] Chen, J. S. et al. Analysis and interpretation of hot springs water, biofilms, and sediment bacterial community profiling and their metabolic potential in the area of Taiwan geothermal ecosystem. *Sci. Total Environ.* **856**, 159115 (2023). 36181827 10.1016/j.scitotenv.2022.159115

[123] Sriaporn, C., Campbell, K. A., Van Kranendonk, M. J. & Handley, K. M. Bacterial and archaeal community distributions and cosmopolitanism across physicochemically diverse hot springs. *ISME Commun.* **3** (1), 80 (2023). 37596308 10.1038/s43705-023-00291-z PMC10439147

[124] Shimuta, T. R. et al. Novel heat shock protein HspQ stimulates the degradation of mutant DnaA protein in *Escherichia coli*. *Genes Cells*. **9** (12), 1151–1166 (2004). 15569148 10.1111/j.1365-2443.2004.00800.x

[125] Kumsta, C. & Jakob, U. Redox-regulated chaperones. *Biochemistry* **48** (22), 4666–4676 (2009). 19368357 10.1021/bi9003556 PMC2848813

[126] Singh, H., Appukuttan, D. & Lim, S. Hsp20, a small heat shock protein of *Deinococcus radiodurans*, confers tolerance to hydrogen peroxide in *Escherichia coli*. *J. Microbiol. Biotechnol.* **24** (8), 1118–1122 (2014). 24743570 10.4014/jmb.1403.03006

[127] Jiao, L., Ran, J., Xu, X. & Wang, J. Heat, acid and cold stresses enhance the expression of DnaK gene in *Alicyclobacillus acidoterrestris*. *Food Res. Int.* **67**, 183–192 (2015).

[128] Sakoh, M., Ito, K. & Akiyama, Y. Proteolytic activity of HtpX, a membrane-bound and stress-controlled protease from *Escherichia coli*. *J. Biol. Chem.* **280** (39), 33305–33310 (2005). 16076848 10.1074/jbc.M506180200

[129] Sangpuii, L. et al. Comparative roles of clpA and clpB in the survival of *S. Typhimurium* under stress and virulence in poultry. *Sci. Rep.* **8** (1), 4481 (2018). 29540723 10.1038/s41598-018-22670-6 PMC5852057

[130] Nagar, S. et al. Microbial ecology of sulfur biogeochemical cycling at a Mesothermal hot spring atop northern Himalayas, India. *Front. Microbiol.* **13**, 848010 (2022). 35495730 10.3389/fmicb.2022.848010 PMC9044081

[131] Konrad, R. et al. Distribution and activity of sulfur-metabolizing bacteria along the temperature gradient in phototrophic mats of the Chilean hot spring Porcelana. *Microorganisms* **11** (7), 1803 (2023). 37512975 10.3390/microorganisms11071803 PMC10385741

[132] Ghilamicael, A. M., Budambula, N. L., Anami, S. E., Mehari, T. & Boga, H. I. Evaluation of prokaryotic diversity of five hot springs in Eritrea. *BMC Microbiol.* **17** (1), 203 (2017). 28938870 10.1186/s12866-017-1113-4 PMC5610464

[133] Das, S., Kumari, A., Sherpa, M. T. & Najar, I. N. Thakur N. Metavirome and its functional diversity analysis through microbiome study of the Sikkim Himalayan hot spring solfataric mud sediments. *Curr. Res. Microb. Sci.* **1**, 18–29 (2020). 34841298 10.1016/j.crmicr.2020.05.002 PMC8610333

[134] Davidson, I. M., Nikbakht, E., Haupt, L. M., Ashton, K. J. & Dunn, P. J. Methodological approaches in 16S sequencing of female reproductive tract in fertility patients: a review. *J. Assist. Reprod. Genet.* **42** (1), 15–37 (2025). 39433639 10.1007/s10815-024-03292-6 PMC11805751

[135] Abellan-Schneyder, I. et al. Primer, pipelines, parameters: issues in 16S rRNA gene sequencing. *Msphere* **6** (1), 10–1128 (2021). 10.1128/mSphere.01202-20 PMC8544895 33627512

[136] Mignard, S. & Flandrois, J. P. 16S rRNA sequencing in routine bacterial identification: a 30-month experiment. *J. Microbiol. Methods*. **67** (3), 574–581 (2006). 16859787 10.1016/j.mimet.2006.05.009

